# The role of anti-EGFR agents in the first-line treatment of advanced esophago-gastric adenocarcinoma: a meta-analysis

**DOI:** 10.18632/oncotarget.20958

**Published:** 2017-09-16

**Authors:** Bum Jun Kim, Jung Han Kim, Hyun Joo Jang, Hyeong Su Kim

**Affiliations:** ^1^ Division of Hemato-Oncology, Department of Internal Medicine, Kangnam Sacred-Heart Hospital, Hallym University Medical Center, Hallym University College of Medicine, Seoul 07441, Republic of Korea; ^2^ Department of Internal Medicine, National Army Capital Hospital, The Armed Forces Medical Command, Seongnam 13574, Republic of Korea; ^3^ Division of Gastroenterology, Department of Internal Medicine, Dongtan Sacred-Heart Hospital, Hallym University Medical Center, Hallym University College of Medicine, Hwasung 18450, Republic of Korea

**Keywords:** esophageal cancer, gastric cancer, anti-EGFR agent, meta-analysis

## Abstract

The role of anti-epidermal growth factor receptor (EGFR) therapy is controversial in patients with esophago-gastric adenocarcinoma. We performed this meta-analysis to evaluate whether the addition of an anti-EGFR agent to chemotherapy can produce survival benefits in patients with advanced esophageal adenocarcinoma, gastric adenocarcinoma, or gastroesophageal junction adenocarcinoma. Electronic databases were searched for eligible randomized studies. From six studies, 1,817 patients were included in the meta-analysis of hazard ratios (HRs) for progression-free survival (PFS) and overall survival (OS). Compared with chemotherapy alone, anti-EGFR agents in combination with chemotherapy were significantly associated with shorter PFS (HR = 1.14 [95% confidence interval {CI}, 1.01–1.28], *P* = 0.03). In terms of OS, the addition of an anti-EGFR agent to chemotherapy showed no advantage (HR = 1.10 [95% CI, 0.98–1.23], *P* = 0.11). In addition, the combination of an anti-EGFR agent with chemotherapy significantly increased some grade 3/4 toxicities including diarrhea (risk ratio {RR} = 1.42, [95% CI, 1.03–1.94], *P* = 0.03), mucositis (RR = 3.30 [95% CI, 1.54–7.07], *P* = 0.002), and skin rash (RR = 6.82 [95% CI, 3.15–14.78], *P* < 0.00001). In conclusion, this meta-analysis indicates that the addition of an anti-EGFR agent to chemotherapy conveys no additional benefit for patients with advanced esophago-gastric adenocarcinoma. As of now, anti-EGFR agents should not be used in the first-line treatment of adenocarcinoma of the upper gastrointestinal tract.

## INTRODUCTION

Gastric cancer and esophageal cancer are one of the common cancers worldwide, in terms of incidence as well as mortality [1, 2]. Radical surgery with or without perioperative or adjuvant treatment offers a potential chance of cure for patients with localized disease; however a considerable number of patients present with advanced disease at the time of diagnosis. Moreover, more than half of the patients treated with complete resection develop recurrence within five years after surgery [3, 4]. For patients with advanced or metastatic diseases, combination chemotherapy can prolong median overall survival (OS) from 3–4 months to approximately 10–13 months with best supportive care [5, 6]. Despite the introduction of new therapeutic regimens, however, the five-year survival rate is still less than 10%; therefore, the development of more efficacious treatments is needed.

Epidermal growth factor receptor (EGFR) is overexpressed in more than 30% of gastric adenocarcinoma (GAC) and esophageal adenocarcinoma (EAC) cases [7, 8]. The overexpression of EGFR and its association with a poor prognosis provide a rationale for the use of anti-EGFR agents in patients with advanced or metastatic EAC or GAC. Anti-EGFR antibodies are molecularly targeted agents that exhibit anti-tumor activity by blocking a cascade of signal transduction pathway. In patients with metastatic colorectal cancer, the addition of targeted agents such as cetuximab and panitumumab to standard chemotherapy significantly improves OS [9, 10]. Then, anti-EGFR therapy has also received a great attention in esophageal or gastric cancer, and various anti-EGFR agents have been tested in randomized controlled trials [11–20]. The addition of an anti-EGFR agent to chemotherapy did not significantly enhance progression-free survival (PFS) and OS in patients with squamous cell carcinoma (SCC) of the esophagus and gastroesophageal junction [11, 12]. However, the role of anti-EGFR therapy is controversial in patients with adenocarcinoma of the esophagus or stomach.

We performed this meta-analysis of randomized trials to evaluate whether the addition of an anti-EGFR agent to chemotherapy can produce survival benefits in patients with advanced EAC, GAC, or gastroesophageal junction adenocarcinoma (GEJAC).

## RESULTS

### Results of search

**Figure 1:**
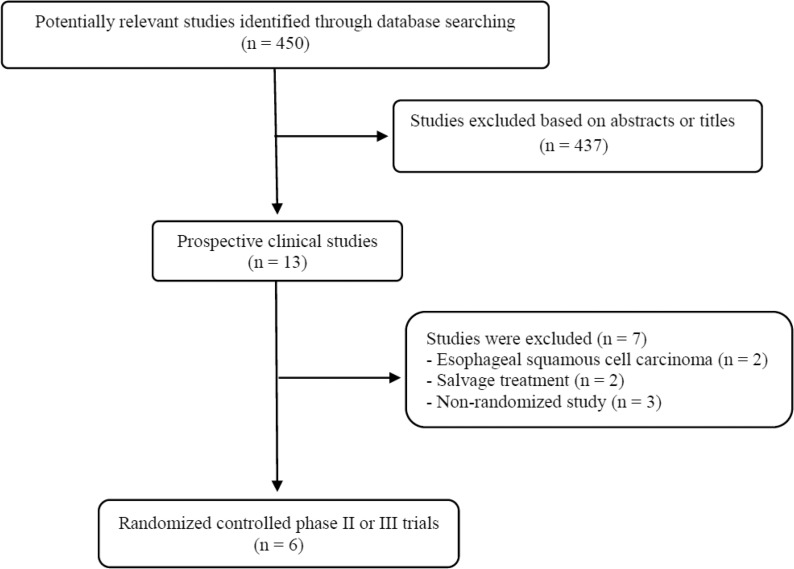
Flow diagram of search process

The flowchart of our study is shown in Figure 1. A total of 450 potentially relevant studies were identified by our searching strategy; 437 were excluded after carefully screening the titles and abstracts. Of the remaining 13 prospective studies, 7 were further excluded by the inclusion criteria: two randomized trials consisted of patients mainly with esophageal SCC [11, 12] and other two conducted in a salvage treatment setting were excluded [13, 14]. Finally, six randomized clinical trials were included in this meta-analysis [15–20].

### Characteristics of the eligible studies

Table 1 summarizes the main characteristics of the six included studies. Three studies were conducted in patients with GAC or GEJAC [16, 17, 19] and the other three in patients with EAC, GAC, or GEJAC [15, 18, 20]. In one study with 77 patients, 5 (6.5%) with esophageal SCC were also included [20]. There were two phase III trials [16, 18] and four phase II trials [15, 17, 19, 20]. The included studies were all conducted in the first-line treatment setting for patients with advanced esophago-gastric cancer. The anti-EGFR agents used included matuzumab [15], cetuximab [16, 17], panitumumab [18, 20], and nimotuzumab [19].

**Table 1:** Summary of the six included studies

Author, trial name (year)	Phase, Setting	Primary site	No. of patients	Treatment arms	Primary endpoint	ORR	mPFS (mo)	HR for PFS (95% CI)	mOS (mo)	HR for OS (95% CI)
Rao et al., (2010)	II 1st-line	E/GEJ/S	35	Epirubicin 50 mg/m^2^ + cisplatin 60 mg/m^2^ on day 1 + capecitabine 1250 mg/m^2^ daily on days 1–21 and matuzumab 800 mg weekly	ORR	31%	4.8	1.13 (0.63–2.01) *P* = 0.678	9.4	1.02 (0.61–1.70) *P* = 0.945
			36	Same without matuzumab		58%	7.1		12.2	
Lordick *et al*., EXPAND (2013)	III 1st-line	GEJ/S	455	Capecitabine 1000 mg/m^2^ on days 1–14 + cisplatin 80 mg/m^2^ on day 1 q3wks + cetuximab 400 mg/m^2^ on day 1 (first), then 250 mg/m^2^ weekly	PFS	30%	4.4	1.09 (0.92–1.29) *P* = 0.32	9.4	1.00 (0.87–1.17) *P* = 0.95
			449	Same without cetuximab		29%	5.6		10.7	
Richards *et al*., (2013)	II 1st-line	GEJ/S	75	Docetaxel 60 mg/m^2^ + oxaliplatin 130 mg/m^2^ on day 1 q3wks + cetuximab 400 mg/m^2^ on day 1 (first), then 250 mg/m^2^ weekly	PFS	38.0%	5.1	0.85 (0.57–1.28) *P* = 0.445	9.4	0.92 (0.64–1.34) *P* = 0.663
			75	Same without cetuximab		26.5%	4.7		8.5	
Waddell *et al*., REAL3 (2013)	III 1st-line	E/GEJ/S	278	Epirubicin 50 mg/m^2^ + oxaliplatin 100 mg/m^2^ on day 1 and capecitabine 1000 mg/m^2^ on days 1–21 + panitumumab 9 mg/kg on day 1 q3wks	OS	46%	6.0	1.22 (0.98–1.52) *P* = 0.068	8.8	1.37 (1.07–1.76) *P* = 0.13
			275	Epirubicin 50 mg/m^2^ + oxaliplatin 130 mg/m^2^ on day 1 and capecitabine 1250 mg/m^2^ on days 1–21 q3wks		42%	7.4		11.3	
Du *et al*., (2015)	II 1st-line	GEJ/S	31	S-1 80mg/m^2^on days 1–14 and cisplatin 30 mg/m^2^ on days 1& 2 q3wks + weekly nimotuzumab 200 mg/m^2^	ORR	54.8%	4.8	2.14 (1.19–3.83) *P* = 0.011	10.2	1.78 (0.97–3.25) *P* = 0.062
			31	Same without nimotuzumab		58.1%	7.2		14.3	
Tebbutt *et al*., ATTAX3 (2016)	II 1st-line	E/GEJ/S	38	Docetaxel 30 mg/m^2^ on days 1 & 8 + cisplatin 60 mg/m^2^ on day 1 + 5-FU 160 mg/m^2^ daily or capecitabine 1000 mg/m^2^ daily q3wks + panitumumab 9 mg/m^2^ on day 1	ORR	58%	6.0	1.14 (0.68–1.91) *P* = 0.614	10.0	1.49 (0.83–2.67) *P* = 0.183
			39	Same without panitumumab		49%	6.9		11.7	

E, esophagus; GEJ, gastroespophageal junction; S, stomach; 5-FU, 5-fluorouracil; ORR, overall response rate; CI, confidence interval; mOS, median overall survival; mPFS, median progression-free survival; HR, hazard ratio; wks, weeks; NA, not available.

### Survival

**Figure 2:**
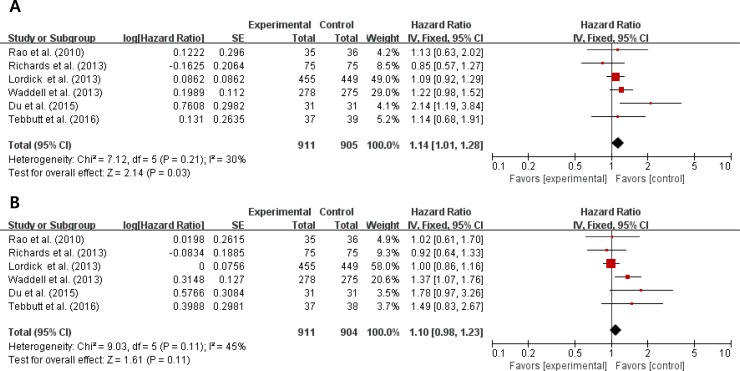
Forest plots of hazard ratios regarding progression-free survival (A) and overall survival (B)

From the six studies [15–20], 1,817 patients were included in the meta-analysis of hazard ratios (HRs) for PFS and OS. Compared with chemotherapy alone, anti-EGFR agents in combination with chemotherapy were significantly associated with a shorter PFS (HR = 1.14 [95% confidence interval {CI}, 1.01–1.28], *P* = 0.03) (Figure 2A). We adopted the fixed-effects model because there was no significant heterogeneity (*X*^2^ = 7.12, *P* = 0.21, *I*^2^ = 30%).

In terms of OS, the addition of an anti-EGFR agent to chemotherapy showed no advantage (HR = 1.10 [95% CI, 0.98–1.23], *P* = 0.11) (Figure 2B). We also used the fixed-effects model because there was no significant heterogeneity (*X*^2^ = 9.03, *P* = 0.11, *I*^2^ = 45%).

### Adverse events

Omitting the REAL3 trial with the different doses of chemotherapeutic drugs between two arms [18], the five studies [15–17, 19, 20] were included in the meta-analysis of adverse events (AEs). Table 2 shows the estimated risk ratios (RRs) of common AEs. The addition of an anti-EGFR agent to chemotherapy significantly increased some grade 3/4 toxicities including diarrhea (RR = 1.42, [95% CI, 1.03–1.94], *P* = 0.03), mucositis (RR = 3.30 [95% CI, 1.54–7.07], *P* = 0.002), and skin rash (RR = 6.82 [95% CI, 3.15–14.78], *P* < 0.00001). Hypokalemia and asthenia tended to occur more frequently when an anti-EGFR agent was added to chemotherapy. Interestingly, however, the addition of an anti-EGFR agent significantly reduced the rate of grade 3/4 neutropenia (RR = 0.68 [95% CI, 0.52–0.89], *P* = 0.004).

**Table 2:** The estimated risk ratio (RR) for common grade 3/4 adverse events

Adverse events	Anti-EGFR agent + chemotherapy			Chemotherapy				*P*	RR (95% CI)
	Total	Events	%	Total	Events	%	*I^2^*		
Neutropenia	597	148	24.8	571	233	40.8	19%	0.004	**0.68 (0.52–0.89)**
Anemia	512	43	8.4	503	51	10.1	0%	0.32	0.81 (0.53–1.23)
Thrombocytopenia	549	25	4.6	535	27	5.0	0%	0.63	0.87 (0.49–1.53)
Febrile neutropenia	144	17	11.8	143	15	10.5	0%	0.75	1.13 (0.53–2.44)
Diarrhea	590	58	9.8	579	33	5.7	0%	0.007	**1.85 (1.18–2.91)**
Mucositis	483	29	6.0	475	9	1.9	0%	0.002	**3.30 (1.54–7.07)**
Vomiting	518	39	7.5	511	42	8.2	0%	0.71	0.92 (0.58–1.44)
Asthenia	590	66	11.2	579	47	8.1	0%	0.07	1.45 (0.97–2.16)
Hypokalemia	481	58	12.1	472	41	8.7	0%	0.09	1.44 (0.95–2.19)
Neuropathy	107	9	8.4	104	7	6.7	0%	0.65	1.28 (0.44–3.67)
Skin rash	590	90	15.3	579	11	1.9	50%	< 0.00001	**6.82 (3.15–14.78)**
Hand-foot syndrome	518	35	6.8	511	16	3.1	66%	0.72	1.29 (0.32–5.29)
Cardiac events	481	31	6.4	472	22	4.7	56%	0.98	1.02 (0.36–2.85)
Death	481	43	8.9	472	36	7.6	0%	0.45	1.19 (0.75–1.89)

### Publication bias

**Figure 3:**
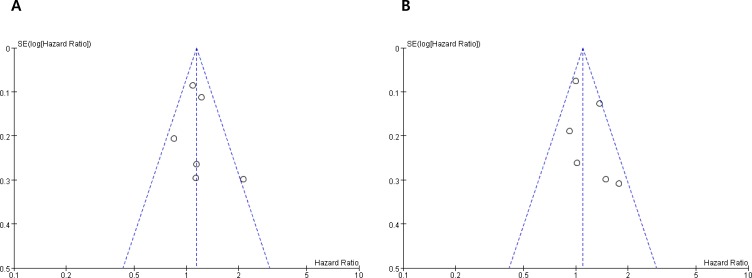
Funnel plots for publication bias test regarding progression-free survival (A) and overall survival (B)

Visual inspection of the funnel plots and Egger's test for PFS (*P* = 0.152) and OS (*P* = 0.087) indicated that there were no substantial publication biases (Figure 3A and 3B).

## DISCUSSION

Several meta-analyses already reported that anti-EGFR combination therapy did not improve PFS and OS in patients with esophago-gastric cancers [21, 22]. However, those studies had major limitations that patients with SCC of the upper esophagus or patients with various treatment settings were included in the analysis. In the current study, we investigated the role of anti-EGFR targeted agents in the first-line treatment setting for patients with advanced/metastatic EAC, GAC or GEJAC. The meta-analysis of six randomized studies revealed that the addition of an anti-EGFR agent to chemotherapy led to no improvement of PFS and OS with a potential risk of increasing severe AEs.

The poor outcomes associated with the addition of an anti-EGFR agent to chemotherapy were not attributable to increased treatment-related deaths; therefore, other potential hypotheses need to be considered. First, the lack of additional benefit of anti-EGFR agents might be in part due to the reduction of dose intensity of chemotherapy in response to AEs. In this meta-analysis, the addition of an anti-EGFR agent significantly increased some grade 3/4 AEs including diarrhea, mucositis, and skin rash. Thus, these severe toxicities may serve to reduce the dose intensity of chemotherapy in the experimental group with an anti-EGFR agent. In the REAL3 study, the addition of panitumumab to chemotherapy (epirubicin, oxaliplatin, and capecitabine) was initially observed to be toxic [18]. After reducing the doses of oxaliplatin and capecitabine in the experimental group, patients receiving panitumumab plus the modified chemotherapy showed worse OS, compared with those in the control group (8.8 vs. 11.3 months, *P* = 0.013). However, there was no significant difference in the dose intensity of chemotherapeutic agents between two arms in other four studies included in this meta-analysis [15, 16, 19, 20]

Second, the absence of efficacy might be attributable to a negative pharmacokinetic interaction between anti-EGFR agents and chemotherapeutic agents [23, 24]. Out of the six studies included in this meta-analysis, four used capecitabine. In a meta-analysis of four randomized studies with colorectal cancer patients, only patients treated with infusional 5-fluorouracil (5-FU)-based chemotherapy derived benefit from cetuximab [23]. Compared with patients treated with infusional 5-FU, those who received capecitabine or bolus 5-FU showed a 42% decrease in response probability (*P* < 0.001) and a 52% (*P* < 0.001) and 33% (*P* = 0.012) increase in the risk of progression and death, respectively. These results suggest that the interactions between anti-EGFR agents and cytotoxic agents may affect their efficacy as well as toxicity.

Third, *KRAS* mutation status can also affect the efficacy of anti-EGFR therapy. In patients with metastatic colorectal cancer, *KRAS* mutation is a negative predictive marker for anti-EGFR therapy. However, the expected frequency of *KRAS* mutation is only approximately 3% in EACs or GACs [25–27]. In this meta-analysis, the relevant data was insufficient to analyze the impact of *KRAS* mutation on drug efficacy because mutational status was assessed only in a small portion of patients.

To increase efficacy and avoid unnecessary toxicities, discovering other predictive biomarkers to identify the correct candidates for anti-EGFR therapy is essential for patients with advanced or metastatic esophago-gastric cancer. Several studies in these cancers have suggested that EGFR expression, EGFR gene copy number, or expression of other EGFR ligands (epiregulin and amphiregulin) might be potential biomarkers for efficacy of anti-EGFR antibodies [27–29]. The EGFR expression level was a predictive marker of survival benefits in advanced non-small-cell lung cancer patients treated with cetuximab and first-line chemotherapy [30]. In the EXPAND study, however, EGFR immunohistochemistry score was not associated with PFS or OS in either treatment group [16]. Further translational studies, with respect to candidate biomarkers, are required before considering anti-EGFR therapy for those patients.

Of note, our study has several limitations. First, the small number of included studies is a major limitation of this meta-analysis. Second, the individual studies enrolled cancer patients with different primary sites (low esophagus, gestroesophageal junction, and stomach). Third, the experimental group used different anti-EGFR agents (catuximab, panitumumab, matuzumab, and nimotuzumab). Finally, as we mentioned above, one study included five patients with esophageal SCC; however, because they occupied only a small portion of patients, inclusion of these patients did not seem to affect the results.

In conclusion, this meta-analysis indicates that the addition of an anti-EGFR agent to chemotherapy conveys no additional benefit for patients with advanced EAC, GAC, or GEJAC. As of now, anti-EGFR antibodies should not be used in the treatment of adenocarcinoma of the upper gastrointestinal tract. Translational studies to explore predictive biomarkers are warranted to identify ideal candidates of anti-EGFR therapy.

## MATERIALS AND METHODS

### Searching strategy

This study was carried out in accordance with the Preferred Reporting Items for Systematic Reviews and Meta-Analyses (PRISMA) guidelines [31]. The electronic databases PubMed, EMBASE, and Clinical Trials (ClinicalTrials.gov) up to December 2016 were systematically searched. We also manually searched the following congress abstract databases: American Society for Clinical Oncology (ASCO) Annual Meeting, ASCO Gastrointestinal Cancers Symposium, and European Society for Medical Oncology (ESMO) Congress. The search was performed using the following terms: “gastric cancer,” “gastroesophageal junction cancer,” “esophageal cancer,” “randomized,” “anti-EGFR or anti-epidermal growth factor receptor,” “cetuximab,” “panitumumab,” “matuzumab,” “nimotuzumab,” “gefitinib,” “erlotinib,” or “vandetanib” in various combinations. The related articles function in PubMed was also used to identify all related articles.

### Inclusion and exclusion criteria

Eligible studies were required to meet the following inclusion criteria: (i) prospective randomized, controlled trials in patients with advanced/metastatic adenocarcinoma of the esophagus, gastroesophageal junction, or stomach; (ii) randomization of chemotherapy-naïve patients to either anti-EGFR agent plus chemotherapy or chemotherapy alone; (iii) HRs and their 95% CIs for PFS or OS were reported or could be calculated from the data provided.

The studies consisting mainly of patients with squamous cell carcinoma of the esophagus were excluded.

### Data extraction

The following data were carefully extracted from all eligible studies: the last name of first author, year of publication, trial phase, the number of participants, treatment regimens, ORR, PFS and OS with their HRs and 95% CIs, and incidence of grade 3 or higher AEs.

Data extraction was done independently by two authors (BJK and JHK). If these two authors could not reach a consensus, other authors (HSK and HJJ) were consulted to resolve the disagreement.

### Statistical analysis

Statistical values used in the meta-analysis were directly obtained from the original articles or were indirectly calculated from the given data. If HR and 95% CI were not reported directly, the Engauge Digitizer version 9.1 was used to obtain the needed data from Kaplan-Meier curves. The effect size of RFS and OS was pooled through HR and its 95% CI. Heterogeneity among studies was estimated using the *I*^2^ inconsistency test and chi-square-based Cochran's Q statistic test, and *P* < 0.1 and *I*^2^ > 50% indicated the presence of significant heterogeneity. The fixed-effects model (Mantel-Haenszel method) was used to calculate the pooled HR and RR when substantial heterogeneity was not observed. When there was a substantial heterogeneity, we adopted the random-effects model (DerSimonian-Laird method). Final results were presented with HR or RR and 95% CI. All reported *P*-values were two-sided, with *P* < 0.05 defined as statistically significant. Statistical analyses were performed using the Review Manager 5.2 software [32]. The possibility of publication bias was assessed with the Egger's test [33] and visual inspection of the funnel plots [34].
